# Some Remarks on Peri-Rectal Suppuration

**Published:** 1909-11-13

**Authors:** 


### SURGERY.

### SOME REMARKS ON PERI-RECTAL SUPPURATION.

It is hardly surprising, when one considers the anatomy of the rectum and its neighbourhood, that suppuration round it should be more or less common. The ischio-rectal fossa is a large cavity filled with a pad of coarse fat, which, though it is fairly well supplied with blood-vessels, has a very low power of resistance against infection. Much stress has been laid on the fact that the process is sometimes set up by an injury of the wall of the bowel. It is true that the hard fæces in being passed may tear down one of the folds of Morgagni, or a foreign body such as a fish-bone may actually perforate the wall of the bowel, thus allowing the escape of the bacillus coli into the adjacent structures. But it is not even necessary to have an antecedent injury for this to occur. In appendicitis the bacillus coli makes its way out through the wall of the appendix without any antecedent injury, when either its virulence is increased or the resistance of the peritoneum is impaired. Identically the same thing may happen in the rectum, and this is the explanation of the fact that in the great majority of cases of ischio-rectal abscess there is no evidence of any damage to the rectal wall itself. What happens is that the patient's general resistance to infection is diminished, as in diabetes or albuminuria, or the local balance is upset as by prolonged sitting on a damp seat, and infection results.

It is true that the infection comes in nearly all cases from the rectum, although the micro-organism responsible is not always the bacillus coli; as often as not a staphylococcus can be cultivated from an ischio-rectal abscess. It may then be asked, what is the path of infection? The infection may occur by direct extension from the rectum without any antecedent damage, or it may arrive via the veins or lymphatics of the part.

It may then occur apparently quite spontaneously, or it may follow one of the following conditions: rectal ulcer, piles, carcinoma, or simple stricture of the rectum. In the latter case it must be regarded simply as a symptom of the more serious condition underlying it.

### Position.

There are three situations in which an abscess may occur: (1) Underneath the skin round the anal margin, superficial to the sphincter. This is a slight malady, and usually heals readily after incision. (2) In the ischio-rectal fossa itself; this is the commonest variety. And (3) by the side of the rectum above the levator ani; this is a serious condition, because there is no upper limit to the abscess, and it may spread up along the intestine and give rise to deep-seated suppuration.

But it is to the ischio-rectal abscess proper and to the importance of treating it adequately that we particularly wish to draw attention. It is not difficult to recognise. The history of pain to one or other side of the rectum, which is exacerbated on defæcation, together with the presence of a red, hot, tender, and possibly fluctuating swelling in the ischio-rectal fossa, should be sufficient to establish the diagnosis.

As soon as its presence is recognised the abscess should be opened. The patient should be anæsthetised and placed in the lithotomy position. One or more incisions should then be made to give exit to the pus. The actual position of these is a matter of small moment, provided that a free exit is given to the pus, so that no pocketing can occur. Perhaps the most serviceable incision is a cruciform one. The entire limits of the abscess cavity should be opened up and explored, and when all the pus has been evacuated, the opening should be lightly packed with gauze, and it should be redressed daily so that it can granulate up from the bottom. If free incisions are made in this way, there may be fairly free and almost alarming hæmorrhage, but this is easily controlled by catching the bleeding points and ligaturing them. It is better to spend a little time over this than to send the patient back to bed with a small vessel still spouting in the depths of the wound, because, when the patient comes round from the anæsthetic, the hæmorrhage may become more profuse, and to arrest the bleeding without an anæsthetic with the patient in bed may be a matter of the greatest difficulty, besides putting him to unnecessary pain and inconvenience.

### Complications.

Improper or insufficient surgical treatment will lead to the formation of a fistula; this result invariably follows if an ischio-rectal abscess is left to pursue its course untreated. The abscess makes its way along the line of least resistance, which may be towards the rectum or towards the surface, and thus a discharging fistula is formed, blind internal or blind external according to the circumstances; but sooner or later the fistula becomes complete, the track leading from the lumen of the rectum to the surface, and finally it may burrow round the rectum, forming the so-called "horseshoe" fistula, and even infect the ischio-rectal fossa of the opposite side.

In the treatment of this, the same precautions must be observed as in ischio-rectal abscess, that is to say the operation must be thorough. A probe pointed director is passed along the track from the external opening into the rectum and out at the anus. All the intervening tissue must be cut through and every track thoroughly opened up and scraped. The cavity, as before, should be lightly plugged with gauze. In one sense a fistula is a more serious affair than an ischio-rectal abscess, because the sphincter ani must be divided; convalescence cannot be complete until this is repaired, because until that time control of the fæces is not regained, a period usually of four to six weeks.

After the operation the bowels should be opened with castor oil on the fourth morning; and it will be found best to let the patient get up for this and expel the plugging in the act of defæcation. The wound can then be plugged again. After the first occasion little or no pain will be caused by this.

